# Dietary fasting and time-restricted eating in Huntington’s disease: therapeutic potential and underlying mechanisms

**DOI:** 10.1186/s40035-024-00406-z

**Published:** 2024-04-02

**Authors:** Russell G. Wells, Lee E. Neilson, Andrew W. McHill, Amie L. Hiller

**Affiliations:** 1 Department of Neurology, Oregon Health and Science University, 3181 SW Sam Jackson Park Rd, Portland, OR 97239 USA; 2 Neurology and PADRECC VA Portland Health Care System, Portland, OR 97239 USA; 3 Sleep, Chronobiology and Health Laboratory, School of Nursing, Oregon Health & Science University, Portland, OR 97239 USA; 4 Oregon Institute of Occupational Health Sciences, Oregon Health & Sciences University, Portland, OR 97239 USA

**Keywords:** Huntington’s disease, Dietary fasting, Intermittent fasting, Time-restricted eating, Lifestyle intervention, Neuroprotection, Autophagy, Mitochondrial biogenesis, Circadian rhythm

## Abstract

Huntington's disease (HD) is a devastating neurodegenerative disorder caused by aggregation of the mutant huntingtin (mHTT) protein, resulting from a CAG repeat expansion in the huntingtin gene *HTT*. HD is characterized by a variety of debilitating symptoms including involuntary movements, cognitive impairment, and psychiatric disturbances. Despite considerable efforts, effective disease-modifying treatments for HD remain elusive, necessitating exploration of novel therapeutic approaches, including lifestyle modifications that could delay symptom onset and disease progression. Recent studies suggest that time-restricted eating (TRE), a form of intermittent fasting involving daily caloric intake within a limited time window, may hold promise in the treatment of neurodegenerative diseases, including HD. TRE has been shown to improve mitochondrial function, upregulate autophagy, reduce oxidative stress, regulate the sleep–wake cycle, and enhance cognitive function. In this review, we explore the potential therapeutic role of TRE in HD, focusing on its underlying physiological mechanisms. We discuss how TRE might enhance the clearance of mHTT, recover striatal brain-derived neurotrophic factor levels, improve mitochondrial function and stress-response pathways, and synchronize circadian rhythm activity. Understanding these mechanisms is critical for the development of targeted lifestyle interventions to mitigate HD pathology and improve patient outcomes. While the potential benefits of TRE in HD animal models are encouraging, future comprehensive clinical trials will be necessary to evaluate its safety, feasibility, and efficacy in persons with HD.

## Background

Huntington’s disease (HD) is an inherited autosomal-dominant neurodegenerative disorder characterized by abnormal movement control (including involuntary choreatic movements, poor fine motor control, and balance impairment), cognitive decline, and psychiatric symptoms [1]. The disease is caused by expansion of a triplet repeat (CAG) in the Huntingtin gene which encodes the huntingtin protein (HTT), where a repeat length of 40 or more is known to eventually cause symptomatic HD and a repeat length of 36–39 is considered incompletely penetrant [2–4]. Penetrance is influenced by a number of factors including somatic expansion of CAG in the brain that is thought to contribute to the rate of disease progression in a length- and tissue-dependent manner [5, 6]. The resulting mutant huntingtin (mHTT) protein induces neuronal dysfunction by disrupting various cellular components and pathways, resulting in oxidative damage, transcriptional dysregulation, excitotoxicity, and bioenergetic deficiencies [7–9]. As the disease progresses, areas outside the brain become affected, leading to atrophy of skeletal muscle, cardiac failure, and dysfunction in the circadian rhythm [10, 11].

Incomplete understanding of the precise functions of wild-type HTT paired with the heterogeneous harmful effects caused by mHTT has made the underlying pathology difficult to target therapeutically. For example, there is a well-known connection between CAG repeat length and age at disease onset at the population level, yet there is much variability in individual symptom timing, progression, and severity [12, 13]. Genome-wide association studies have highlighted DNA repair mechanisms, mitochondrial redox factors, and CAA alleles as major contributors to this variability [14, 15]. Additionally, environmental factors and lifestyle habits are thought to also modify the age of onset and the severity of disease [16, 17]. Identifying and addressing these lifestyle habits could hold therapeutic potential in delaying HD progression.

Pharmacological interventions often target a very specific aspect of disease or physiologic process. Because mHTT has effects on so many areas of function, lifestyle interventions that can affect multiple processes and pathways may be more beneficial and are worthy of investigation. Growing evidence suggests that a form of intermittent fasting (IF) known as time-restricted eating (TRE)—defined by consuming all daily caloric intake within a 6–8-h time window and fasting the remainder of the 24-h day—may attenuate the progression of neurodegenerative diseases [18–21]. TRE may be particularly well-suited for use in the HD population because of its ability to induce autophagy and clear mHTT, upregulate Sirtuin 1 (SIRT1) and stimulate brain-derived neurotrophic factor (BDNF) production, improve mitochondrial bioenergetics and prevent oxidative stress via mediation of peroxisome proliferator-activated receptor gamma coactivator 1-alpha (PGC-1α), and regulate circadian function. In this review, we explore the role of TRE as a dietary intervention for HD. Here, we highlight all published articles directly related to the effects of TRE in HD, summarize the underlying physiologic mechanisms associated with the diet, and examine its therapeutic potential in HD pathology and symptomology.

## Methods

The literature search was performed in PubMed, MEDLINE, and Google Scholar databases with the following keywords: “Huntington’s disease”, “time-restricted eating”, “time-restricted feeding”, “dietary fasting”, “dietary restriction”, “intermittent fasting”, “fasting”, “fast/feed cycle”, “circadian modulation”, and/or “circadian rhythms”. Abstracts were reviewed, and articles that examined the effects of any form of dietary fasting in the context of HD (animals or humans) were included after the full-text was retrieved (Table 1). References of the included studies were also screened. Studies on fasting in non-HD models were excluded.

**Table 1:** Summary of studies exploring the effects of dietary fasting and TRE in HD

Study	Models/patients	Intervention	Fasting/feeding time per cycle	Results
Duan et al. [85]	N171-82Q HD mice	3-month ADF	24-h/24-h	Delayed disease onset; slowed disease progression; ↑ survival; ↑ motor function; ↓ brain atrophy; ↓ mHTT aggregate formation and apoptotic protease activation; normalized blood glucose regulation; ↓ tissue wasting and weight loss; ↑ BDNF and protein chaperone levels in brain
Ehrnhoefer et al. [79]	YAC128 HD mice	1-week TRE	18-h/6-h	↓ mTOR; ↑ SIRT1; ↑ neuronal autophagy; ↓ cortical mHTT protein
Wang et al. [130]	Q175 HD mice	3-month TRE	18-h/6-h	↑ Circadian locomotor activity; ↑ coordination in onset of sleep; ↑ HRV; ↑ motor function; restoration of HD-relevant markers in striatal gene expression analysis
Whittaker et al. [131]	BACHD mice	3-month TRE	18-h/6-h	↑ Circadian locomotor activity; ↑ time spent sleeping during rest phase; ↑coordination in onset of sleep; ↑ HRV; ↑ motor function
Phillips et al. [113]	N-of-1, unblinded clinical trial of a 41-year-old male patient with HD	48-week TRKD	2 meals/day, 1 h/meal, no snacks	Improvements in motor symptoms, activities of daily living, cUHDRS score, behavioral problems, irritability, mood-related quality of life; no change in cognition; weight remained stable, no reported adverse effects

*HD* Huntington’s disease; *N171-82Q* transgenic mouse model of HD that expresses the N-terminal 171 amino acids of human huntingtin protein with 82 polyglutamine repeats; *YAC128* transgenic mouse model of HD that contains a full-length human huntingtin gene modified with a 128 CAG repeat expansion; *Q175* transgenic mouse model of HD that contains human huntingtin gene exon 1 sequence with ~ 179 CAG repeats; *BACHD* transgenic mouse model of HD that expresses full-length human huntingtin gene modified to contain *loxP-*flanked human mutant huntingtin exon 1 sequence with 97 mixed CAA-CAG repeats; *ADF* alternate day fasting; *TRE* time-restricted eating; *TRKD* time-restricted ketogenic diet; *mHTT* mutant huntingtin protein; *BDNF* brain-derived neurotrophic factor; *mTOR* mechanistic target of rapamycin; *SIRT1* sirtuin 1; *HRV* heart rate variability; *cUHDRS* composite unified Huntington’s disease rating scale; ↑: increase; ↓: decrease

## HD pathogenesis

HD is a trinucleotide repeat expansion disease caused by a mutant form of the HTT protein. HTT is produced ubiquitously in the human body and although its function has not been fully elucidated, it is reported to be involved in transcriptional regulation, cell morphology and cytoskeleton function, the immune system, cellular metabolism, synaptic function, and antiapoptotic activity [22–24]. The resulting mHTT from a CAG repeat greater than 36 is thought to cause HD-specific pathology in striatal GABAergic spiny projection neurons (SPNs). The neurotoxic effects of the protein lead to cell death and subsequent frontostriatal circuitry malfunction that produce hyperkinetic then hypokinetic motor symptoms as the disease progresses [25]. Although the primary pathology is regionally specific, eventually all areas of the brain become affected, contributing to the cognitive, motor, and psychiatric symptomology observed in HD.

The exact mechanism of HD pathophysiology is not fully known; however, a hallmark of HD is the presence of mHTT aggregates and inclusion bodies inside cells [26]. In vitro*, *in vivo, and postmortem human HD brain studies have identified age-dependent accumulation of striatal intranuclear and cytoplasmic mHTT inclusions [27–29]. Controversially, some in vitro models do not form inclusion bodies; however, further exploration suggests that the brain microenvironment and the aging process may be required for mHTT inclusion formation [30–32]. These inclusion bodies are thought to play a role in the neuronal dysfunction and cell death found in HD and are a primary target for therapeutic intervention where the prevention of *HTT* gene expression or improved clearance of mHTT aggregates both serve as theorized potential treatments for the disease. With recent advances in gene therapy technology, many trials have attempted to lower mHTT in persons with HD; however, unfortunately all trials to date have failed to significantly improve clinical outcomes. Reasons for this are speculative and not fully understood. Likely contributing factors include somatic expansion and the fact that the breadth of system dysfunction in HD is difficult to collectively address with targeted pharmacologic therapies.

An additional contributing factor to the pathophysiology of HD is the deficiency of BDNF in the striatum. BDNF is an essential protein involved in the survival and proliferation of neurons in the peripheral and central nervous systems. Specifically, BDNF produced from cortical pyramidal neurons is required for proper corticostriatal synaptic connections and survival of GABAergic SPNs [33]. BDNF has also been found to have neuroprotective effects against the NMDA-dependent excitotoxicity, a hypothesized contributing factor to HD pathogenesis [34]. Substantial decreases in striatal BDNF levels observed in HD are thought to contribute to the onset and severity of motor dysfunction [33, 35]. Restoration of these levels may have therapeutic potential, though several drawbacks to direct BDNF replacement exist: proper administration requires an invasive procedure, control over gene expression in vivo is limited, epileptogenic risk may be increased, and potentially harmful neural effects may be seen in humans which have not been observed in models with short lifespans [36–39].

The pathogenesis of HD is further convoluted by disturbances in cellular metabolism and oxidative damage. Studies of post-mortem HD brains, adult HD skeletal muscles, and human HD cell lines have revealed ubiquitous bioenergetic dysregulation, indicative of the meaningful role HTT plays in mitochondrial energy metabolism [40, 41]. Post-mortem HD brain analysis reveals significant glycolytic and electron transport chain enzyme activity deficiencies, in addition to increased levels of oxidative damage in the caudate and putamen [42]. Growing evidence suggests that mHTT can inhibit mitochondrial function directly, and indirectly via aberrant transcriptional regulation [43]. The expression of PGC-1α, a key transcriptional co-factor involved in mitochondrial biogenesis, cellular respiration, and reactive oxygen species (ROS) detoxification, is thought to be a potential link between transcriptional dysregulation and mitochondrial dysfunction in HD [44, 45]. Clinically, metabolic dysregulation and bioenergetic imbalance have been associated with lower body weight and body mass index primarily observed via reduced bone mineral density and variably decreased lean body mass and truncal fat in persons with premanifest and manifest HD [46–48]. There may be sex-dependent differences which need to be explored [48]. While weight loss is associated with the clinical progression of HD, it is important to note that body weight is not found to causally affect disease progression [49]. mHTT interference of the mitochondrial respiratory chain is thought to be at least in part responsible for a reduced anaerobic threshold observed in persons with HD where muscle wasting and compensatory increases in caloric intake are commonly seen. This resulting shunt to glycolysis and lactic acid production is expected to accelerate muscle atrophy and disease severity [41, 50].

Attenuation of aberrant cellular metabolic processes involved in HD remains a potential target for therapeutic intervention [46–48]. Many preclinical studies have explored various metabolic interventions that have shown promise, although none to date have translated to an efficacious treatment in humans with HD, highlighting the vast differences in metabolism between rodents and humans [51–54]. Considering the complex pathogenesis of HD, it is evident that either a combinatorial therapeutic approach or a singular treatment strategy capable of attenuating multiple underlying components of the disease is desperately needed.

## Models of fasting

Dietary fasting is a broad term used to describe when a person does not take in any calories during a certain time of the day, the week, or the month. Fasting protocols in humans can take many different forms and are typically exercised because of religious beliefs, cultural practices, health-seeking behavior, or popular dietary trends [55]. IF, also referred to as intermittent metabolic switching and intermittent energy restriction, is a type of dietary fasting that involves alternating between eating and fasting on a regular schedule. Some common schedules include the 5:2 diet, where eating is unrestricted 5 days of the week and fasting is practiced two days of the week; the alternate day fasting, where fasting is practiced every other day; and TRE, where fasting for a specific number of hours, usually greater than 12 and commonly between 16 and 18, is practiced daily [55, 56]. Additionally, TRE protocols can vary depending on the timing of the eating window, frequently described as early-TRE (eTRE) (e.g., eating from 8:00 am to 2:00 pm) or delayed-TRE (dTRE) (e.g., eating from 12:00 pm to 8:00 pm) [57]. Recent literature has begun to delineate the benefits and challenges of each approach. In an animal study that directly compared eTRE and dTRE, both featuring high-fat diets, the two schedules equally improved glucose tolerance; however, weight gain and insulin resistance were higher in the dTRE group [58]. Another similar study of high-fat diets found that although rats in each group consumed a similar number of calories, the body weight gain was higher and markers of the circadian clock were delayed in the dTRE group [59]. When comparing the two approaches in adult men with obesity, similar improvements in glucose tolerance were observed between dTRE and eTRE [60]. Future studies are needed to further elucidate differences between the two TRE approaches both in practicality and in the effect on health.

It is important to note that dietary fasting is different from caloric restriction (CR), which is defined by a chronic and sustained reduction in energy intake without incurring malnutrition [61, 62]. CR has been well studied and is associated with longevity in preclinical studies and various health benefits in humans; however, the practice has proven to be exceedingly challenging to sustain as a lifestyle intervention, with reported clinical dropout rates reaching as high as 40% in some trials [61, 63]. Although caloric intake may be reduced with fasting, the majority of studies described below and mechanisms underlying the health benefits of fasting are achieved when caloric intake remains equivalent to normal ad libitum diets. The focus, therefore, lies on the metabolic switch that occurs between states of energy consumption and fasting.

## Underlying mechanisms of fasting in HD

**Fig. 1:**
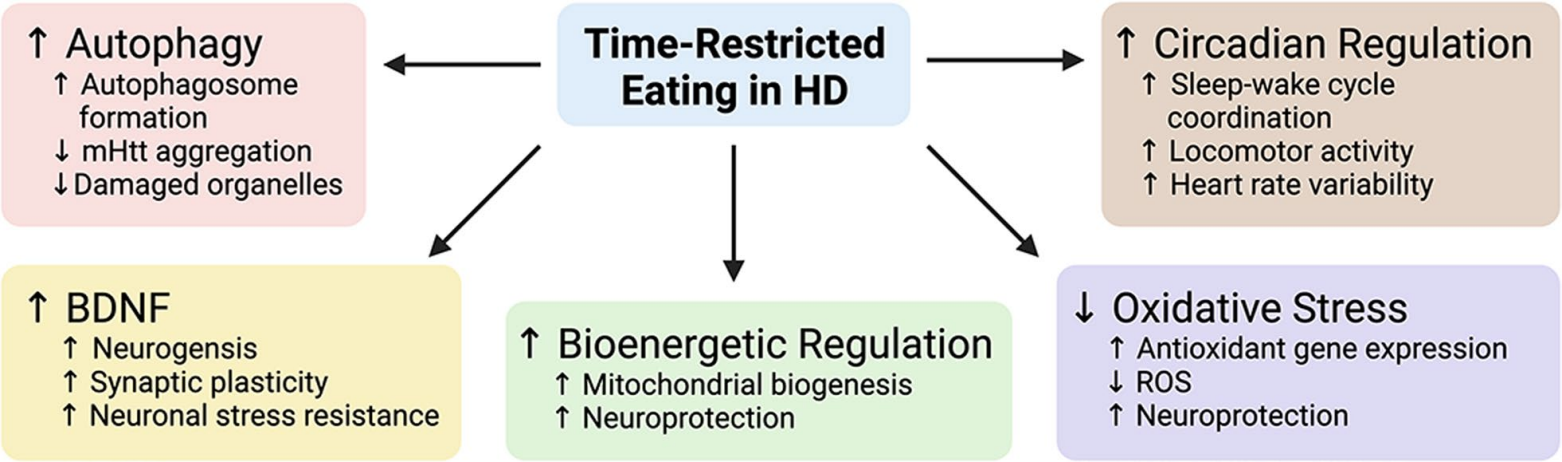
Model of mechanisms underlying the therapeutic potential of time-restricted eating in Huntington’s disease (HD). Scheduled daily eating and fasting, known as time-restricted eating (TRE), in HD and non-HD human and animal studies reveals that the practice increases autophagic activity which is thought to decrease aggregation of the mutant huntingtin protein (mHTT), stimulates production of brain-derived neurotrophic factor (BDNF), improves metabolic functions, promotes oxidative stress resistance, decreases reactive oxygen species (ROS), and improves measures of circadian rhythm function. Created with BioRender.com

**Fig. 2:**
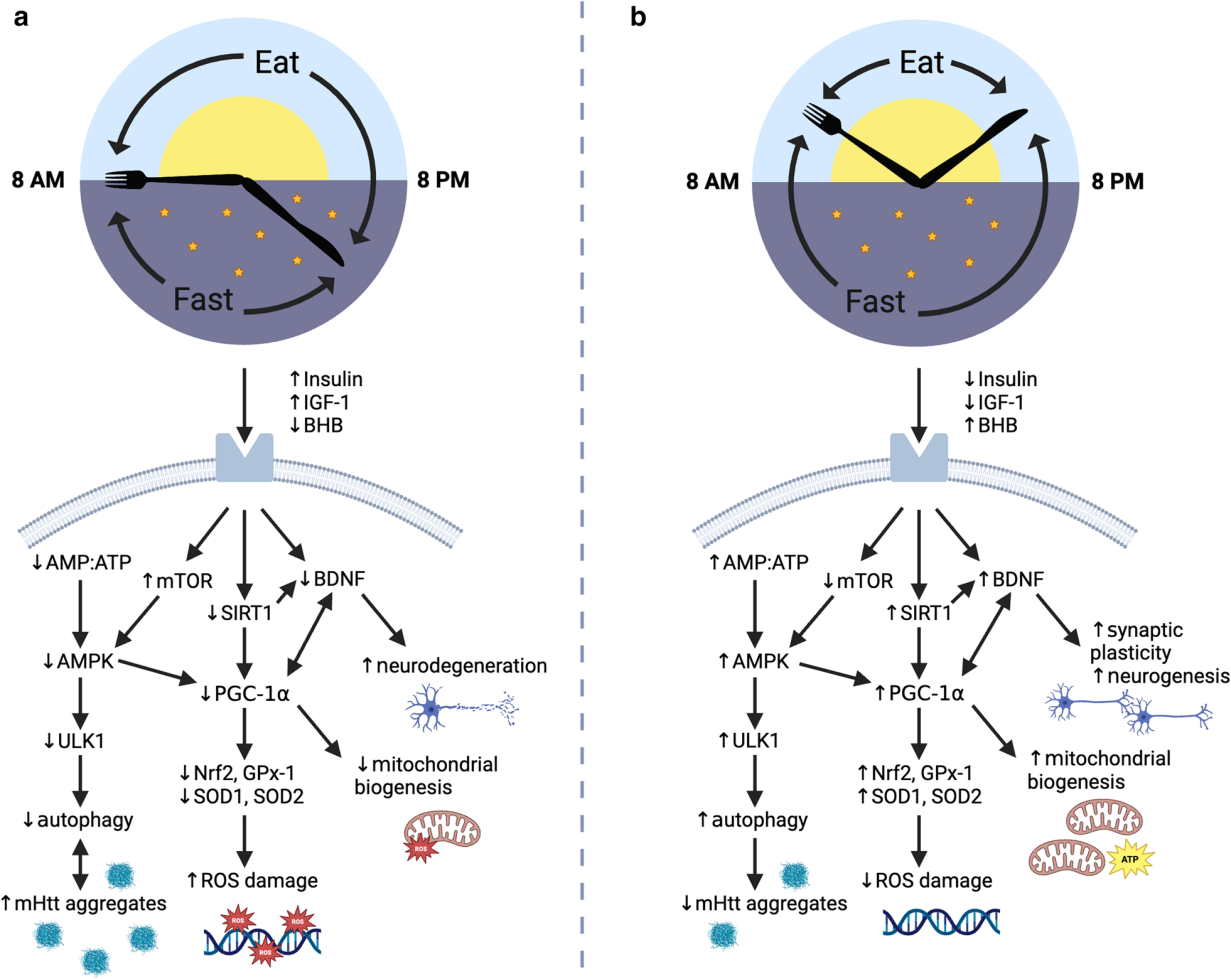
Potential neuroprotective effects mediated by time-restricted eating in Huntington’s disease (HD). **a** Chronic caloric exposure increases levels of insulin and IGF-1 and decreases the cellular AMP/ATP ratio, leading to activation of mTOR and downregulation of AMPK activity, respectively. As a result, autophagic processes are not stimulated and mHTT protein aggregates accumulate, which further inhibit cellular autophagic activity. Decreased SIRT1 in a fed state and decreased BDNF expression seen in HD pathology result in downregulation of PGC-1α and subsequent oxidative stress, neurodegeneration, and metabolic dysregulation. **b** Fasting in TRE downregulates mTOR and upregulates AMPK, which stimulate autophagy through ULK1 activation. Increased autophagy is known to reduce mHTT aggregate formation in neurons. Fasting in TRE also upregulates SIRT1, which has been shown to potentiate PGC-1α and promote oxidative stress resistance and mitochondrial biogenesis. States of fasting additionally result in increased peripheral blood levels of BHB which upregulate BDNF expression. BDNF induces neurogenesis and synaptic plasticity and activates PGC-1α for further metabolic regulation and antioxidant effects. Created with BioRender.com

The exact biochemical and physiologic mechanisms underlying all the health benefits associated with dietary fasting remain to be fully elucidated. However, mounting evidence over the past few decades has uncovered many noteworthy findings. Specific data from preclinical and clinical studies reveal that TRE may have implications in HD onset and progression. Here, we highlight mechanisms associated with IF and TRE that may hold therapeutic interest: clearance of mHTT, recovery of striatal BDNF levels, improvement of mitochondrial and stress-response function, and circadian rhythm regulation (Figs. 1 and 2).

### Autophagy and mHTT clearance

Maintenance of proper cell function, especially of permanent cells such as neurons, depends heavily on the cellular autophagic system, a degradation pathway responsible for the removal of damaged proteins and organelles, protein complexes and aggregates, and pathologic substances [64, 65]. Deficiencies in autophagy have been associated with a host of diseases such as viral and bacterial infections, cancer, and specific neurodegenerative diseases characterized by protein aggregation [66–69]. A multifactorial dysregulation and dysfunction of the autophagic system has been reported in cellular and rodent models of HD and in human HD tissues. This is believed to contribute to the accumulation of damaged organelles and mHTT aggregates that result in subsequent neurotoxicity [70–72]. Both soluble and aggregated forms of mHTT are expected to be degraded preferentially through autophagy, therefore restoration of the cellular mechanism holds therapeutic potential [69, 73]. Several studies have demonstrated that pharmacologic induction of the autophagic system leads to attenuation of mHTT aggregation and a reduction in mHTT-mediated toxicity in cell and animal HD models [70, 74, 75].

It is well-recognized that fasting is a potent stimulator of autophagy. At the molecular level, both the mechanistic target of rapamycin (mTOR) and AMP-activated protein kinase (AMPK) play a key role in mediating autophagic activity. States of prolonged energy restriction result in decreased insulin and IGF-1 levels that diminish mTOR activity, and an increased AMP/ATP ratio that stimulates AMPK. This combination induces autophagy by activating the mammalian autophagy-initiating kinase ULK1 [76]. In mice, fasting has been shown to dramatically upregulate neuronal autophagy as evidenced by increased abundance of autophagosome formation and decreased neuronal mTOR activity [77]. A two-week TRE intervention in healthy humans revealed that serum mRNA expression levels of ULK1 and other proteins involved in autophagy induction were significantly increased and remained elevated for the duration of the restricted feeding schedule [78].

The effects of TRE and IF have recently been explored in HD animal models (Table 1). In the YAC123 transgenic HD mouse model, 24-h fasting induced autophagy that resulted in significant reductions in mHTT protein levels in the liver, an organ that relies on autophagy for basal function [79]. Further analysis revealed that mRNA expression of the mutant *HTT* transgene in fasted mice was equivalent to that in the non-fasted mice, suggesting that fasting does not affect gene expression and that autophagic degradation is likely responsible for mHTT protein clearance. The same study also examined cortical brain tissue in YAC123 mice after one-week TRE consisting of an 18-h fasting and 6-h feeding schedule. Compared to mice with ad libitum access to food, the TRE schedule downregulated mTOR activity, increased the overall number of neuronal autophagic vesicles, and reduced cortical mHTT protein levels. The findings suggest that TRE has the potential to increase the clearance of mHTT protein in the mammalian brain through fasting-induced autophagy and indicate a need for future studies in human subjects and persons with HD to explore the translation and clinical relevance of these physiologic effects.

### BDNF recovery

TRE and IF are well-documented stimulants for the expression of BDNF, a protein mediator of brain function involved in neurogenesis, synaptic plasticity, and neuronal stress resistance [80]. When in a fasted state, glycogen stores are depleted, and fatty acids are released into the blood for energy use and ketone body production. It is thought that one specific ketone, β-hydroxybutyrate (BHB), acts as a molecular signal to induce BDNF expression in neurons as one of the brain’s natural stress-response mechanisms [81]. Recent data reveal that BHB can directly upregulate BDNF expression in neurons of the cerebral cortex and hippocampus in cell culture and in vivo [82, 83]. Many preclinical studies in animal models of Alzheimer’s disease and Parkinson’s disease consistently support the ability of TRE and IF to increase levels of BDNF in the mammalian brain [84]. When applied to the N171-82Q transgenic HD mouse model, alternate day fasting for 3 months significantly increased levels of BDNF in the striatum and cerebral cortex by 3–4 folds compared to transgenic mice maintained on an ad libitum diet. Interestingly, wild-type mice maintained on the IF diet in the same study also showed a 3–4-fold increase in BDNF levels when compared to wild-type mice eating ad libitum [85]. The study additionally found that IF in transgenic mice, when compared to those on an ad libitum diet, delayed HD symptom onset, slowed disease progression, improved survival and motor function, reduced brain atrophy, decreased mHTT aggregate formation and apoptotic protease activation, normalized blood glucose levels, and prevented tissue wasting and weight loss. It is expected that these benefits arise from restoration of striatal BDNF levels paired with processes of upregulated autophagy, improved metabolic regulation, and cytoprotective oxidative stress resistance.

### Bioenergetic regulation and oxidative stress response

Mitochondrial biogenesis is a process by which cells produce new mitochondria, often in response to metabolic challenges and transcription factors that promote cellular stress resistance. PGC-1α, AMPK, and SIRT1 are key regulators of mitochondrial biogenesis, a process severely hindered in HD [86–88]. When looking at striatal neurons from moderate-to-severe grade HD patients, there are significant grade-dependent reductions in the number and density of mitochondria that correlate with marked decreases in PGC-1α expression [89]. Analysis of striatal neurons from persons with HD, knock-in HD animal models, and HD cell lines reveals significant reductions in PGC-1α mRNA expression and target genes [45, 90]. Knockdown of PGC-1α in neuronal cell cultures not only inhibits mitochondrial biogenesis, but also eliminates the ability of BDNF to promote synaptogenesis [91]. Upregulating PGC-1α can additionally enhance BDNF expression, and it is thought that the two proteins exhibit a bidirectional positive feedback relationship [92]. AMPK, acting as a metabolic sensor responsive to an increased AMP/ATP ratio, is also thought to directly activate PGC-1α, thereby potentiating its mitochondrial regulatory role in maintaining metabolic homeostasis [93]. Brain tissues from persons with HD also exhibit decreased SIRT1 mRNA and protein levels [94, 95]. SIRT1 is thought to promote cell survival and mediate important neuroprotective processes in part through its ability to upregulate BDNF expression [96]. Studies in the N171-82Q and BACHD transgenic mouse models of HD display that overexpression of SIRT1 improves motor function, prevents cortical and striatal atrophy, and attenuates neuronal cell death in the striatum [97, 98]. The polyphenol, resveratrol, has displayed neuroprotective properties in HD animal models through activation of SIRT1 and PGC-1α, resulting in ameliorated mHTT peripheral tissue damage, improved motor coordination, and reduced striatal atrophy [51, 99, 100].

The numerous interactions of PGC-1α are further expanded by its key role in cellular oxidative stress-response signaling. PGC-1α suppresses ROS and activates mitochondrial antioxidant cascades through increased expression of nuclear factor erythroid 2-related factor 2 (Nrf2), copper/zinc superoxide dismutase, manganese SOD (SOD2), catalase, and glutathione peroxidase GPx-1 [101, 102]. Nrf2 is a transcription factor that has recently gained specific attention in neurodegenerative therapeutic research due to its role in modulating expression of antioxidant genes and regulation of mitochondrial function [103–105]. Overexpressing PGC-1α in HD transgenic mice ameliorates motor dysfunction and neurodegeneration by reducing oxidative stress and mHTT aggregates [106]. In a transgenic HD rat model, overexpression also increases neuronal mitochondrial density and cell survival [107].

Emerging evidence suggests that IF and TRE stimulate mitochondrial biogenesis and oxidative stress-resistance. When fasting, increases in BHB and the ratio of AMP/ATP stimulate release of BDNF and activate AMPK, respectively. Expression of SIRT1 is also elevated in response to the metabolic challenge. As a result, BDNF, AMPK, and SIRT1 each serve to potentiate PGC-1α for maintenance of cellular bioenergetic regulation and oxidative stress resistance. IF in *C. elegans* promotes longevity and mitochondrial homeostasis through AMPK activation [108]. In diabetic mice, fasting every other day induces mitochondrial biogenesis through upregulation of SIRT1/AMPK signaling and activation of PGC-1α in the liver [109]. IF in diabetic mice improves cognitive function through enhanced mitochondrial biogenesis and energy metabolism gene expression, specifically increased PGC-1α and phosphorylated AMPK, in the hippocampus [110]. Importantly, the aforementioned studies established these findings in models unrelated to HD and it remains to be known if the same processes would be observed in the context of HD pathology. That said, TRE for one week, with daily fasting periods of 18 h and eating periods of 6 h, does result in significantly increased mRNA levels of SIRT1, a known PGC-1α stimulant, in the cortex of YAC128 transgenic HD mice [79].

While data on fasting and bioenergetic regulation in persons with HD are yet to be explored, a prospective study of 56 overweight and obese subjects engaging in Ramadan IF, consisting of daily fasting for roughly 15 h for one month, resulted in significantly increased expression of the antioxidant genes *SOD2* and *Nrf2* [111]. A recent 36-month longitudinal study of 99 older adults with mild cognitive impairment compared subjects who fasted regularly twice a week from sunrise to sunset to those who did not fast, and found that the regular fasting group exhibited significant increases in SOD activity, decreased C-reactive protein levels, decreased markers of DNA damage, and improved cognitive status from baseline to follow-up [21]. Another study found that 5 weeks of TRE (18-h fast/6-h feed) improved insulin sensitivity, blood pressure, and oxidative stress in men with prediabetes [112]. Recently, and intriguingly, a case study in a 41-year-old male HD patient with progressively deteriorating symptoms, reported that a 48-week combined metabolic strategy of TRE paired with a ketogenic diet resulted in improvements of motor symptoms, activities of daily living, composite Unified HD Rating Scale (UHDRS) score, and psychiatric symptoms [113]. Since this is a case study, it is not possible to precisely determine the mechanism of the improvements. While a placebo effect or improved control of the participant's diabetes could help explain the effect, the authors hypothesized that there was enhanced brain and muscle energy metabolism and mitochondria function, possibly through upregulation of PGC-1α. No biochemical measures were undertaken, but it has set the stage for a small single-arm, open-label trial on ketogenetic diets in HD (ID ACTRN12620000281998) [114].

The accumulating data indicate a potential clinical application for TRE in HD; however, further knowledge is needed to better understand the underlying mechanisms and the efficacy in mediating bioenergetic function and oxidative stress resistance in persons with HD.

### Circadian regulation

A less studied, yet common characteristic of HD is circadian rhythm disturbance and sleep–wake cycle dysfunction. Exemplified by disruptions in sleep and shifted sleep timing that worsen with disease severity and correlate with poor cognitive performance and psychiatric symptoms, many persons with premanifest and manifest HD experience increased sleep onset latency, decreased sleep quality, abnormal sleep architecture and efficiency visualized by polysomnography, increased daytime sleepiness, and later wake times [115–118]. Transgenic mouse models of HD also display similar sleep–wake cycle and circadian dysfunction that is thought to be related to the pathophysiologic function of the suprachiasmatic nucleus (SCN) in the hypothalamus, the brain’s principal circadian clock, that results in progressively declining rhythm amplitudes and disruptions in circadian regulation of heart rate and body temperature [119, 120]. The Q175 knock-in model of HD reveals gene dosage- and age-related degradation of circadian rhythms, observed through deficits in locomotor activity, suggestive of a relationship between HD pathology and circadian disruption [121]. However, the exact mechanism underlying circadian dysfunction and sleep–wake disturbance observed in persons with HD remains to be fully understood. Nonetheless, it is well known that insufficient duration of quality sleep has a negative impact on daily living for all persons, including those who are neurologically healthy. The sleep and circadian abnormalities observed in HD are assumed to contribute to symptom severity and potentially disease progression and are hence a target for therapeutic intervention [122].

The SCN in the hypothalamus is the central clock that drives the body’s rhythms in alertness, feeding, and body temperature. Environmental light conditions serve as the primary temporal cue for circadian regulation that is mediated by the SCN. Additional clocks with influence on the body’s sleep–wake cycle also exist in peripheral tissues where cellular circadian rhythms are intimately related to metabolic pathways [123]. Because of this, data primarily from animal studies suggest that meal timing entrains the circadian clock through tissue-specific regulation [124]. Controlling the feeding rhythm specifically through TRE can coordinate circadian synchronization by regulating peripheral clocks [125]. This is thought to be an underlying mechanism in studies wherein TRE results in improved sleep quality and duration in overweight human subjects and patients with metabolic syndrome [126, 127].

While no studies examining TRE and circadian modulation have been conducted in persons with HD, mouse models of HD have revealed progressive, age-dependent SCN impairment that can potentially be circumvented through peripheral clock regulation by TRE as evidenced by its benefit in SCN-lesioned mice [128, 129]. The effects of 3-month TRE (18-h fast/6-h feed during the middle of the active phase) on circadian function in the Q175 and BACHD mouse models of HD have also revealed many notable findings. Compared to transgenic mice on an ad libitum diet, both studies found that TRE-treated mice experienced improved measures of locomotor activity, sleep onset and awakening time, heart rate variability, and motor performance [130, 131]. These data support the need for further exploration in persons with HD where circadian-based treatment strategies could serve to delay HD progression and improve quality of life.

## Potential adverse effects and important considerations

Considering the existing data on TRE in animals and humans, it is apparent that the diet may promote adaptive cellular responses responsible for a range of neurologic health benefits. However, applying a lifestyle habit that has been associated with weight loss is a primary safety concern in a disease like HD, which is known for causing weight loss especially in later stages of disease [46]. It is reassuring, however, that a study evaluating the safety and tolerability of TRE (16-h fast/8-h feed) in healthy midlife and older adults for six weeks found that the diet had no influence on body mass, lean mass, bone density, or nutrient intake, although it is important to note that the subjects were advised by a dietitian on how to maintain healthy daily caloric consumption within the restricted eating window [132]. Another randomized controlled trial in young men performing resistance training found that eight weeks of TRE (20-h fast/4-h feed 4 days/week) reduced energy intake but did not adversely affect lean mass or muscle performance [133]. Additionally, it is noteworthy that weight loss associated with IF and TRE is primarily found in studies with overweight and obese subjects [134]. It is also interesting that alternate day fasting for three months in HD transgenic mice reduced the amount of tissue wasting and weight loss when compared to mice eating ad libitum [85]. These data may be related to an adaptive response of increased growth hormone release in states of fasting that exists to protect muscle mass and vital organ functions [135].

Persons with premanifest and manifest HD have also been found to consume a higher number of calories when compared to age-matched controls, which is thought to be related to increased involuntary muscle activity and energy expenditure and/or a hypermetabolic state [136, 137]. Given the limited timeframe in TRE where all daily calories should be consumed, it could be challenging for some individuals to successfully maintain the diet. Moreover, because the later stages of HD are associated with significant cognitive impairment, another consideration is that a strict TRE diet may be difficult to adhere to, and for this reason may be more practical in persons that are premanifest or in the early stages of disease.

Additionally, the optimal timing of TRE induction remains to be fully understood where e-TRE (eating breakfast and lunch) may maximize specific metabolic and circadian benefits; however, d-TRE (eating lunch and dinner) may align better with biologic variations in hunger and social schedules that could support mental and emotional well-being [138].

Another consideration is that the healthful effects associated with TRE in non-HD human studies are in the context of functional mitochondria. Future work is needed to better understand how the dietary strategy will affect HD patients who have known mitochondrial dysfunction from early life onwards. It is possible that states of fasting may force metabolism to try and generate ATP increasingly from aerobic glycolysis, a process that normal aging brains are found to avoid and one that is thought to worsen neurodegenerative pathology due to increased ROS stress [139, 140]

It is also worth emphasizing that, although fasting habits have revealed great health potential, the dietary strategy may have limited efficacy, specifically in neurodegenerative diseases, if it is not combined with adequate consumption of highly nutritious foods that are associated with neurologic health and longevity [141, 142]. For example, recent literature has uncovered an association between gut dysbiosis and HD pathogenesis, highlighting the importance of promoting healthy gut microbiota through dietary management, probiotic consumption, and avoidance of ultra-processed foods [143–145]. In addition, lipid homeostasis, which depends on diet composition, appears to be compromised in HD, where sphingolipids may serve as mediators of cellular stress response and HD pathology [146]. Consumption of foods high in healthy unsaturated fats, omega-3 fatty acids, fiber, and antioxidants, and low in refined sugars and saturated fats, such as the Mediterranean diet, may be associated with improved clinical outcomes in HD and could potentially augment the benefits of TRE [141]. Considering the multifactorial metabolic deficits present in HD and the resulting risks of muscle wasting, the maintenance of body composition and skeletal muscle mass depends heavily on sufficient caloric and, more specifically, protein intake—an important consideration if TRE is applied to persons with HD [147]. Notably, the rodent studies highlighted in this review focused on TRE and did not explore the effects of specific dietary composition on measured outcomes, which is a logical next step. Lastly, given the metabolic defects characteristic of HD, it is difficult to generalize findings on fasting benefits from healthy populations to HD, hence why clinical studies are exceedingly necessary.

## Conclusions and future directions

Considering the available data, it seems the time is right to try this intervention in the HD population. The first step will need to be a robust assessment of the safety and feasibility of the diet. It will be imperative to determine if persons with HD can consistently adhere to the diet while maintaining body weight before long-term trials can assess the efficacy of TRE as a potential lifestyle treatment strategy for disease modification. Future studies will also need to explore the optimal schedule of fasting and feeding and investigate the type of food consumption that will be most advantageous. The therapeutic potential of TRE may be additionally augmented when paired with a diet that is associated with improved neurologic health such as the Mediterranean diet. If proven safe, it is reasonable to suspect that the TRE’s potential neuroprotective effects may provide more benefit to persons in earlier stages of disease where underlying pathologic processes may be halted before significant later stage neuronal damage and striatal atrophy set in. Further exploration and clinical analysis of TRE in HD in the near future will hopefully clarify its potential to serve as a viable therapeutic option for persons living with this debilitating disease.

## Data Availability

Data sharing is not applicable to this article as no datasets were generated or analyzed during the current study.

## Abbreviations

AMPK: AMP-activated protein kinase
BACHD: Transgenic mouse model of HD that expresses full-length human huntingtin gene modified to contain loxP-flanked human mutant huntingtin exon 1 sequence with 97 mixed CAA-CAG repeats
BDNF: Brain-derived neurotrophic factor
BHB: β-Hydroxybutyrate
CR: Calorie restriction
HD: Huntington’s disease
HTT: Huntingtin protein
IF: Intermittent fasting
mHTT: Mutant huntingtin protein
mTOR: Mechanistic target of rapamycin
N171-82Q: Transgenic mouse model of HD that expresses the N-terminal 171 amino acids of human huntingtin protein with 82 polyglutamine repeats
Nrf2: Nuclear factor erythroid 2-related factor 2
PGC-1α: Peroxisome proliferator-activated receptor gamma coactivator 1-alpha
Q175: Transgenic mouse model of HD that contains human huntingtin gene exon 1 sequence with ~ 179 CAG repeats
ROS: Reactive oxygen species
SCN: Suprachiasmatic nucleus
SIRT1: Sirtuin 1
SOD2: Manganese superoxide dismutase
SPN: Spiny projection neuron
TRE: Time-restricted eating
YAC128: Transgenic mouse model of HD that contains a full-length human huntingtin gene modified with a 128 CAG repeat expansion
